# The Effects of Metabolic Work Rate and Ambient Environment on Physiological Tolerance Times While Wearing Explosive and Chemical Personal Protective Equipment

**DOI:** 10.1155/2015/857536

**Published:** 2015-03-19

**Authors:** Joseph T. Costello, Kelly L. Stewart, Ian B. Stewart

**Affiliations:** School of Exercise and Nutrition Sciences and Institute of Health and Biomedical Innovation, Queensland University of Technology, Kelvin Grove, QLD 4059, Australia

## Abstract

This study evaluated the physiological tolerance times when wearing explosive and chemical (>35 kg) personal protective equipment (PPE) in simulated environmental extremes across a range of differing work intensities. Twelve healthy males undertook nine trials which involved walking on a treadmill at 2.5, 4, and 5.5 km·h^−1^ in the following environmental conditions, 21, 30, and 37°C wet bulb globe temperature (WBGT). Participants exercised for 60 min or until volitional fatigue, core temperature reached 39°C, or heart rate exceeded 90% of maximum. Tolerance time, core temperature, skin temperature, mean body temperature, heart rate, and body mass loss were measured. Exercise time was reduced in the higher WBGT environments (WBGT37 < WBGT30 < WBGT21; *P* < 0.05) and work intensities (5.5 < 4 < 2.5 km·h^−1^; *P* < 0.001). The majority of trials (85/108; 78.7%) were terminated due to participant's heart rate exceeding 90% of their maximum. A total of eight trials (7.4%) lasted the full duration. Only nine (8.3%) trials were terminated due to volitional fatigue and six (5.6%) due to core temperatures in excess of 39°C. These results demonstrate that physiological tolerance times are influenced by the external environment and workload and that cardiovascular strain is the limiting factor to work tolerance when wearing this heavy multilayered PPE.

## 1. Introduction

Personal protective equipment (PPE) is required in sporting and occupational settings to protect the wearer from a range of hazards [1]. Unfortunately, PPE may increase the rate of metabolic heat production at rest and during exercise. Concomitant elevations in thermoregulatory and cardiovascular strain during exercise in high ambient temperatures and humidity can lead to progressive increases in body heat content and if left unchecked this may lead to heat related illnesses [2]. In humans, the primary source of heat dissipation during exercise is through increased skin blood flow and sweating [2–4]. Wearing PPE may impede evaporative heat loss through sweating and a condition of uncompensable heat stress may occur [5, 6]. Consequently, information regarding work tolerance and rest cycles is of paramount importance for the health of the wearer in an occupational setting.

Several studies have examined the physiological strain encountered by fire fighters [7], police offices [8, 9], security guards [10, 11], pilots [12], and military personnel [13, 14] where PPE is a necessity. The major focus of this research has been the development of safe occupational guidelines for participants wearing PPE in the workplace. This is of particular importance as symptoms of heat illness ranging from headache to loss of consciousness and even death have previously been reported in emergency first responders and military personnel [15–17]. However, there has been comparatively little research done to represent the physiological strain experienced by explosive ordinance disposal (EOD) technicians.

We have previously examined the physiological effects of wearing EOD PPE in the field [17, 18] and in a controlled laboratory [19] setting. However, in theatre EOD technicians regularly have to don chemical PPE in addition to the EOD ensemble when the severity or type of threat is unknown. Typically clothing which confers protection from chemical threats is fully encapsulating and impermeable in nature and requires the use of a respirator or self-contained breathing apparatus [1, 5, 6, 20]. In preparation for such operational scenarios, these technicians regularly train and operate while wearing these ensembles in extreme environments. It is well established that, in isolation, multiple layers of protective clothing, load-carriage, and the use of a respirator have a negative effect on ventilatory function, thermoregulation, and exercise tolerance during prolonged submaximal exercise [1, 13, 21]. Multiple clothing layers and load-carriage increase the energy cost of physical activity, apart from the added weight of the clothing per se [22]. Each layer of protective clothing also traps air between the skin and/or other clothing layers, and a microenvironment which serves as an insulator is created [23, 24]. Moreover, chemical protective garments typically have a high water vapour resistance and this further reduces the ability of the wearer to evaporate sweat [1, 25]. Respirators are also known to impair exercise capacity [21] by placing extra stress on the cardiorespiratory system during exercise. Ultimately, these components combine to increase an individual's metabolic rate and reduce their ability to dissipate heat during exercise, and a condition of uncompensable heat stress is created. Taken together, these findings suggest that the addition of chemical PPE to an EOD ensemble may impair thermotolerance and increase the risk of heat related illness.

Thus, the purpose of the present investigation was to examine the physiological tolerance times while wearing chemical and explosive protective clothing concurrently across a range metabolic work rates and ambient environments. Establishing this information has implications for determining safe tolerance times for EOD technicians when required to wear this PPE in various environments.

## 2. Methods

### 2.1. Participants

Twelve healthy, recreationally active males [(mean ± SD): age = 24.1 ± 3 years, height = 1.79 ± 0.06 m, body mass = 76.4 ± 8.4 kg, body surface area 2.0 ± 0.1 m^2^, sum of eight skinfolds 79.1 ± 31.6 mm, maximal oxygen uptake (${\dot{\text{V}}\text{O}}_{2\max}$) 56 ± 5 mL·kg^−1^·min^−1^, heart rate max 195 ± 9 beats·min^−1^] volunteered to participate in the study. The study was approved by the university human research ethics committee and all participants completed an informed consent form and medical history questionnaire. To eliminate the confounding influences of gender on physiological responses to heat stress, only nonacclimatised, nonsmoking males, free from any known cardiovascular, metabolic, and respiratory diseases, were considered. Participants were asked to refrain from vigorous exercise and avoid the consumption of caffeine and alcohol during the 24 hours preceding the laboratory visits.

Participants attended the laboratory on four separate occasions, at the same time of day, separated by a minimum of 7 days. In the initial visit height and nude body mass were recorded and body surface area was subsequently calculated [26]. Skinfold thickness measures were obtained, using Harpenden (John Bull, West Sussex RH15 9LB, UK) callipers, at eight sites (biceps, triceps, subscapular, iliac crest, supraspinale, abdominal, front thigh, and medial calf). ${\dot{\text{V}}\text{O}}_{2\max}$ was determined by indirect calorimetry during a progressive incremental running protocol on a motorised treadmill [27]. Participants were also provided with the opportunity to familiarise to the PPE ensemble by walking around the laboratory and on the treadmill at the speeds to be utilised for the trials.

### 2.2. Experimental Protocol

In the three remaining laboratory visits participants wore a fully encapsulating NFPA 1994 Class 3 chemical/biological protective garment, including outer gloves and booties (Extended Response Suit, Lion Apparel, Regency Park 5942, South Australia, Australia; 1.35 kg), and a respirator (Promask with a pro2000 PF10 filter; Scott Safety, Lancashire, England; 0.7 kg). The garment was made from trilaminate, a three-layer chemical/biological protective fabric, consisting of a selectively permeable barrier film laminated between outer and inner textiles. A Med-Eng EOD9 suit (Allen Vanguard, Ogdensburg, New York, USA) consisting of a jacket, trousers, groin protection, and a helmet (33.4 kg) was donned over the chemical PPE and respirator. The combined weight of the ensemble was 35.45 kg. Participants' base layer consisted of a t-shirt, shorts, socks, and underwear [28]. Athletic shoes with a soft rubber sole were also worn during testing [28].

During the trials the participants walked on a treadmill, while wearing the PPE, in an environmental chamber (4 × 3 × 2.5 m; length, width, height, resp.). A wet bulb globe temperature (WBGT) of 21, 30, or 37°C was obtained by the following ambient temperatures and relative humidities: 24°C, 50%; 32°C, 60%; and 48°C, 20%; respectively. A simulated wind speed equivalent to ~4.5 km·h^−1^ and a radiant heat load (two radiant heaters positioned ~1.3 m from the participant) were incorporated throughout the testing. These environmental conditions were also monitored independently (Quest Temp, Airmet, Australia). During each of these laboratory visits the participant completed three treadmill-walking trials of 2.5, 4, and 5.5 km·h^−1^ with a 1% gradient. This equated to an external work rate [29] of ~135, 207, and 307 W·m^−2^ for a 76 kg individual with a body surface area of 2.0 m^−2^. The order of the testing, for both the work rate and the environment, was randomised using a random number generator in a controlled crossover design.

During each trial, standard termination criteria were applied in accordance with the American Society for Testing and Materials guidelines [28]: (1) core body temperature reaching 39.0°C; (2) 60 minutes of exercise; (3) heart rate > 90% of maximum; or (4) fatigue or nausea. Following the attainment of one of the termination criteria, the participant exited the environmental chamber and doffed the EOD protective clothing. Participants were then instructed to rest in an air-conditioned room. In the following recovery period participants were provided with food and fluid to a volume equivalent to 125% of the body mass loss in the preceding trial. This was undertaken to ensure recovery of body mass and hydration status prior to commencement of subsequent trials. Core temperature and heart rate were monitored and following their return to baseline levels the participant provided a blood sample, had their nude body mass assessed, and commenced donning the EOD protective clothing for the subsequent trial. Three trials were conducted in this manner per testing session.

### 2.3. Outcome Measures

The primary outcome measure of the current study was physiological tolerance times measured to the nearest 0.5 min. Core temperature was recorded using an ingestible sensor (CorTemp, HQ Inc., Palmetto, FL, USA) swallowed ~6 hours before each trial. This was to allow sufficient time for the sensor to pass from the stomach to the intestines, where the reading of core body temperature is optimal [30]. Weighted mean skin temperature (Tsk) was calculated using four thermochrons (iButton, Maxim Integrated, CA, USA) attached to the back of neck, inferior border of right scapula, dorsal right hand, and proximal third of the right tibia [31]. Mean body temperature was calculated using the equation proposed by Stolwijk and Hardy [32]. Participants also wore a heart rate monitor (Polar Team2, Kempele, Finland) that was attached before entering the environmental chamber. Physiological strain index (PSI) using simultaneous measurements of core temperature and heart rate was calculated using Moran's [33] equation. PSI was rated on a scale of 1–10, with five indicating moderate, seven high, and nine very high physiological strain [33]. Core temperature, skin temperature, mean body temperature, heart rate, and PSI were recorded continuously and averaged over 30 second intervals for data analysis.

Pretrial hydration status was confirmed using urine specific gravity (USG, PAL 10s, ATAGO, Tokyo, Japan) of <1.020. If participants did not meet the above guidelines they were given an additional 500 mL of water to be consumed prior to commencement of the trial. Nude body mass was undertaken following towel drying and measured to the nearest 50 g (Tanita BWB-600, Wedderburn, Australia) before and after each trial. A cannula was inserted in the antecubital fossa for the attainment of venous blood samples in five mL serum separating vacutainers for the determination of serum osmolality using the freezing point depression technique (Osmomat 030, Gonotec, Berlin, Germany) as previously described [19, 34]. Serum osmolality was calculated in duplicate and the coefficient of variation was <1%.

### 2.4. Statistical Analysis

The data are displayed as mean ± SD unless otherwise stated. Assumption of normal distribution of data was assessed using descriptive methods (skewness, outliers, and distribution plots) and inferential statistics (Shapiro-Wilk test). When the assumption of sphericity was violated, significance was adjusted using the Greenhouse-Geisser method to adjust the degrees of freedom to increase the critical values of the *F*-ratio. Tolerance times, body mass loss, and the final value recorded for core temperature, skin temperature, mean body temperature, heart rate, and PSI were analysed using a two-way (environment × work intensity) repeated measures analysis of variance (ANOVA). To determine if baseline physiological and hydration indices were similar, pretrial heart rate, mean body temperature, serum osmolality, and body mass were also analysed in a similar manner. The effect of environment, work intensity, and their interaction were tested. Paired *t*-tests, using a Bonferroni correction, were conducted where significant differences were observed. All data was analysed using SPSS (SPSS version 21.0, SPSS Inc., Chicago, USA). Significance was set* a priori* at the *P* < 0.05 level.

## 3. Results

### 3.1. Baseline Data

Participants commenced all nine trials from a resting physiological baseline (Table 1), with no significant differences between trials in heart rate (*P* = 0.213), mean body temperature (*P* = 0.176), serum osmolality (*P* = 0.407), or body mass (*P* = 0.894). The mean ± SD (range) duration of rest was 91 ± 18 min (58–155) when multiple trials were performed on the same day.

### 3.2. Tolerance Times and Termination Criteria

All twelve participants completed all nine trials (total trials: 108) with no serious adverse events recorded. The majority of trials (85/108; 78.7%) were terminated due to participants' heart rate exceeding 90% of their maximum (Table 2). A total of eight trials (7.4%) lasted the full duration of 60 min. Finally, nine (8.3%) trials were terminated due to volitional fatigue and six (5.6%) due to core temperatures in excess of 39°C.

**Figure 1:**
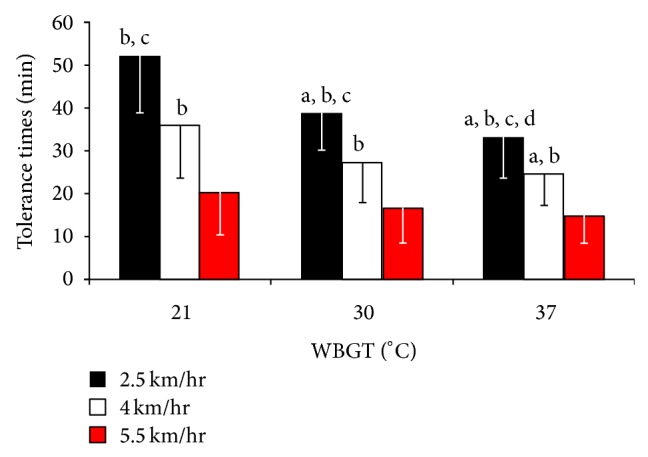
Tolerance time ± SD across the different environmental conditions and work rates (*n* = 12). Main effect observed for environment (WBGT37 < WBGT30 < WBGT21; *P* < 0.001), work intensity (5.5 < 4 < 2.5 km·h^−1^; *P* < 0.001), and their interaction (*P* < 0.001). ^a^Significantly different to the same speed at WBGT 21°C (*P* < 0.05); ^b^significantly different to 5.5 km·h^−1^ at the same environmental condition (*P* < 0.05); ^c^significantly different to 4 km·h^−1^ at the same environmental condition (*P* < 0.05); ^d^significantly different to the same speed at WBGT 30°C (*P* < 0.05).

A significant main effect in tolerance time (Figure 1, Table 2) was observed for environment (WBGT37 < WBGT30 < WBGT21; *P* < 0.001; 1 − *β* = 1.0), work intensity (5.5 < 4 < 2.5 km·h^−1^; *P* < 0.001; 1 − *β* = 1.0), and their interaction (*P* < 0.001; 1 − *β* = 0.999).

### 3.3. Physiological Data

Work intensity (Table 3) had a significant effect on core temperature (*P* < 0.001; 1 − *β* = 0.992), skin temperature (*P* = 0.002; 1 − *β* = 0.936), mean body temperature (*P* < 0.001; 1 − *β* = 0.997), heart rate (*P* = 0.022; 1 − *β* = 0.682), and body mass loss (*P* < 0.001; 1 − *β* = 1.0). Core temperature (*P* < 0.01), skin temperature (*P* < 0.05), and mean body temperature (*P* < 0.01) were lower at the end of the 5.5 km·h^−1^ trials compared to the 2.5 and 4 km·h^−1^ trials. Body mass loss was also greater in the lower work intensities (5.5 < 4 < 2.5 km·h^−1^; *P* < 0.01). Conversely, despite a trend for an increase in heart rate at the 2.5 km·h^−1^ trials compared to the 4 (*P* = 0.055) and 5.5 (*P* = 0.077) km·h^−1^ trials, no post hoc differences were observed.

Skin temperature (*P* < 0.001; 1 − *β* = 1.0), heart rate (*P* = 0.022; 1 − *β* = 0.682), and body mass loss differed across the three environments. Skin temperature (*P* < 0.001) and body mass loss (*P* = 0.027) were significantly higher in the WBGT21 condition compared to the WBGT37 environment. Skin temperature was also higher (*P* = 0.019) in the WBGT21 condition compared to the WBGT30. Heart rate was higher (*P* = 0.02) in the WBGT21 environment compared to the WBGT37 environment. The ambient environment had no significant effect on core temperature (*P* = 0.886; 1 − *β* = 0.056), mean body temperature (*P* = 0.067; 1 − *β* = 0.533), or PSI (*P* = 0.519; 1 − *β* = 0.144). No other statistically significance differences were observed.

## 4. Discussion

The current study is the first to examine the physiological effects of wearing explosive and chemical PPE across a range metabolic work rates and ambient environments. The main findings of this study are that (1) physiological work tolerance is significantly influenced by the external environment and workload, (2) despite the short durations of exercise (~24 min), on average moderate to very high levels of physiological strain were experienced by the participants, and (3) cardiovascular, rather than thermoregulatory, strain is the limiting factor to work tolerance when wearing this ensemble.

As anticipated, tolerance time was reduced in the higher ambient environment and work intensities (Table 2; Figure 1) when wearing the EOD and chemical PPE. However, the ambient temperature and vapor pressure had far less impact on physiological tolerance time as the metabolic rate increased. When the metabolic rate exceeds 250 W·m^−2^ or 500 W, as evident in the 5.5 km·h^−1^ trials, the role the environment plays in the rate of heat storage and work tolerance is limited [5]. These data compliment the findings of Cheung and colleagues [5] and demonstrate minimal differences between the tolerance times in the highest work intensity (>300 W·m^−2^) across the three environments (20.4, 16.9, and 15.1 min in the WBGT21, -30 and -37 environments, resp.; Table 2). In contrast, significant differences were observed in the lower work intensities, especially in the 2.5 km·h^−1^ trials, and tolerance times were greater in the cooler environments (53.1, 39.1, and 33.5 min; Table 2). The actual tolerance times in the WBGT21 environment, when walking at 2.5 km·h^−1^, are likely to be even greater compared to the other conditions as 8 of the 12 participants completed the maximum duration of 60 min (Table 2). These individuals were physically capable of exercising beyond the termination criteria of 60 min; however, work beyond this duration is unlikely when wearing this PPE in the field [17, 18].

The current study is in agreement with previous research findings examining PPE of similar weight [1, 19] and further indicates that cardiovascular strain governs physiological tolerance times regardless of environment or work intensity. Over 78% of the trials in the current study were terminated based on heart rates in excess of 90% of maximum. These near maximal heart rates resulted in moderate to very high levels of physiological strain in almost all trials (Table 3), despite core temperature only reaching 39°C in eight of the 108 trials. The unique finding of this study is that, on average, tolerance times were more than 8 min shorter when wearing the chemical and EOD PPE in comparison to the EOD ensemble in isolation [19]. Although this duration may appear insignificant, it equates to a reduction of more than 20% in exercise tolerance which may be of practical importance for operational success in the field. It is possible to compare these two data sets as the methodologies and the participants were similar between studies. Although a unique group of participants performed both studies, we employed the same methodological protocol and utilised the same environmental conditions (WBGT21, -30, and -37), work rates (2.5, 4, and 5.5 km·h^−1^), and termination criteria [28]. Moreover, the participant demographics and fitness levels (~57 mL·kg^−1^·min^−1^) were similar.

Considering the combined weight of the chemical protective clothing and respirator is minimal (~2 kg); it is unlikely that the additional weight alone was responsible for this reduction in work capacity. For example, Teitlebaum and Goldman [22] have previously demonstrated that metabolic rate increases by only ~3% per layer of clothing. Increased levels of thermal and cardiovascular strain have however been attributed to covering of the head with a helmet [14] or respirator and hood [13] during similar thermotolerance trials. Breathing through a respirator when fully encapsulated also reduces the core temperature that is tolerated at exhaustion by ~0.3°C [13]. This may be related to the effects of breathing through a respirator on the cardiopulmonary system and thermal perception. Respirators, regardless of their make or function [1], typically increase inspiratory and expiratory breathing resistance, decrease maximal voluntary ventilation [21], and reduce ${\dot{\text{V}}\text{O}}_{2\max}$ [35]. Moreover, it is widely known that the head, in particular the forehead, has one of the highest sweat gland densities and usually has a greater sweating response than all other body segments during thermal loading [36, 37]. Consequently, higher mean skin temperatures and greater subjective discomfort are often associated with the use of the mask and breathing through the respirator [13]. This hypothesis is supported in the current study as maximal mean skin temperatures (>39.0°C in some individuals; Table 3) were higher in all trials, despite being 20% shorter in duration, compared to those observed in the EOD ensemble in isolation [19].

It has been postulated that the impact of multiple clothing layers, particularly if associated with extra load carriage, may have greater effects on work tolerance than the added resistance of breathing through a respirator [1, 21]. Regardless of the individual or combined effects of these aforementioned factors, the ensemble utilised in the current study incorporates a plethora of elements that are likely to impair thermotolerance. These include multiple layers of heavy PPE, a helmet, a respirator, and a fully encapsulating suit, examined across a range metabolic work rates and ambient environments. EOD technicians should therefore be cognisant that physiological tolerance times are significantly reduced when a respirator and chemical clothing are added to the EOD PPE.

The findings of this study are limited to young healthy males. Due to physiological differences between males and females [38] and younger and older individuals during exercise in the heat [39], additional research on older and female participants is warranted. The participants in this study also started each trial from a rested physiological state which may not be feasible in the field. Therefore, future research should examine the effects of prior exercise, dehydration, and elevated body temperatures when wearing this type of PPE.

## 5. Conclusions

In conclusion, this investigation has demonstrated that physiological tolerance times are significantly reduced in higher ambient environments and workloads when wearing explosive and chemical PPE. Secondly, despite the short durations of exercise, high to very high levels of physiological strain were experienced by all participants. Finally, cardiovascular strain is the limiting factor to work tolerance when wearing this heavy, multilayered, and encapsulating PPE.

## Figures and Tables

**Table 1:** Baseline physiological and hydration indices (*n* = 12).

Speed (km·h^−1^)	HR (bpm)	*T*_mb_ (°C)	Serum osmolality (mOsmol·kg^−1^)	Body mass (kg)
2.5	102 ± 4.7	36.5 ± 0.08	291 ± 1	76.7 ± 2.26
4	103 ± 4.1	36.5 ± 0.06	292 ± 1	76.7 ± 2.29
5.5	99 ± 3.9	36.4 ± 0.08	292 ± 1	76.7 ± 2.26

Values are means ± SEM. HR, heart rate; bpm, beats per minute; *T*_mb_, mean body temperature.

**Table 2:** Tolerance time (mean, range) and termination criteria across the different environmental conditions and work rates (*n* = 12).

WBGT (°C)	Speed (km·h^−1^)	Tolerance time (min)	HR (>90% max)	*T*_*c*_ (>39°C)	Fatigue or nausea	Duration (=60 min)
21	2.5	52.1 (27.5–60)^b,c^	4			8
	4	36.0 (18–53)^b^	8	1	3	
	5.5	20.4 (6.5–39)	11	1		

30	2.5	39.1 (18.5–51.5)^a,b,c^	9	2	1	
	4	27.4 (12–47.5)^b^	11		1	
	5.5	16.9 (9–28.5)	12			

37	2.5	33.5 (13.5–44.5)^a,b,c,d^	8	1	3	
	4	24.7 (9–33)^a,b^	11	1		
	5.5	15.1 (6–25.5)	11		1	

Values are mean (range). Main effect observed for environment (WBGT37 < WBGT30 < WBGT21; *P* < 0.001), work intensity (5.5 < 4 < 2.5 km·h^−1^; *P* < 0.001), and their interaction (*P* < 0.001). WBGT, wet bulb globe temperature; HR, heart rate; *T*_*c*_, core temperature. ^a^Significantly different to the same speed at WBGT 21°C (*P* < 0.05); ^b^significantly different to 5.5 km·h^−1^ at the same environmental condition (*P* < 0.05); ^c^significantly different to 4 km·h^−1^ at the same environmental condition (*P* < 0.05); ^d^significantly different to the same speed at WBGT 30°C (*P* < 0.05).

**Table 3:** Physiological data at the cessation of each trial (*n* = 12).

WBGT (°C)	Speed (km·h^−1^)	HR (bpm)	*T*_*c*_ (°C)	*T*_sk_ (°C)	*T*_mb_ (°C)	PSI	Body mass loss (%)
21	2.5	164.0 (132–187)^b^	38.3 (37.7–39.0)	37.1 (36.5–38.1)	38.0 (37.5–38.7)	6.7 (4.7–9.2)	1.4 (0.6–2.1)
	4	174.6 (152–187)	38.3 (37.7–39.1)	37.2 (36.8–37.9)	38.1 (37.5–38.9)	7.0 (5.5–8.7)	1.1 (0.5–2.2)
	5.5	178.3 (164–190)	37.9 (37.5–39.0)	36.7 (34.8–38.5)	37.7 (37.2–38.9)	6.6 (5.2–9.0)	0.7 (0.2–1.3)

30	2.5	174.2 (130–186)	38.4 (37.7–39.1)	38.0 (37.4–38.6)	38.3 (37.7–39.0)	7.2 (3.6–9.1)	1.2 (0.6–1.7)
	4	175.0 (135–186)	38.3 (37.6–38.9)	37.9 (37.5–38.5)	38.2 (37.6–38.7)	7.1 (6.0–8.1)	1.0 (0.3–1.5)
	5.5	178.0 (165–188)	37.9 (36.7–38.5)	37.5 (36.7–38.3)	37.8 (36.7–38.5)	6.7 (5.7–8.1)	0.7 (0.3–1.2)

37	2.5	170.7 (113–187)	38.5 (37.9–39.1)	38.5 (38.0–39.2)	38.5 (38.0–39.2)	7.3 (3.6–9.0)	1.2 (0.5–1.7)
	4	178.8 (166–190)	38.2 (37.8–39.3)	38.4 (37.7–39.3)	38.3 (37.8–39.3)	7.2 (5.9–9.5)	0.9 (0.4–1.6)
	5.5	179.2 (166–191)	37.8 (37.4–38.7)	38.0 (36.6–39.4)	37.9 (37.3–38.8)	6.5 (5.4–8.3)	0.6 (0.3–1.1)

Values are mean (range). WBGT, wet bulb globe temperature; HR, heart rate; bpm, beats per minute; *T*_*c*_, core temperature; *T*_sk_, skin temperature; *T*_mb_, mean body temperature; PSI, physiological strain index; ^b^significantly different to 5.5 km·h^−1^ at the same environmental condition (*P* < 0.05). *Note*. Significant main effects (*P* < 0.05) for work intensity (*T*_*c*_, *T*_sk_, *T*_mb_ and body mass loss) and environment (*T*_sk_, HR, body mass loss) were observed—see Results section for main effect comparisons.
